# Identification of nonserotypeable *Shigella* spp. using genome sequencing: a step forward

**DOI:** 10.4155/fsoa-2017-0063

**Published:** 2017-07-07

**Authors:** Muthuirulandi Sethuvel Dhiviya Prabaa, Devanga Ragupathi Naveen Kumar, Inbanathan Francis Yesurajan, Shalini Anandan, Walia Kamini, Veeraraghavan Balaji

**Affiliations:** 1 Department of Clinical Microbiology, Christian Medical College, Vellore – 632 004, India; 2 Division of Epidemiology & Communicable Diseases, Indian Council of Medical Research, New Delhi – 110 029, India

**Keywords:** IncF plasmid, k-mer, nonserotypeable *Shigella*, O-antigen gene cluster, whole genome sequencing

## Abstract

**Aim:**

Sequencing technology has replaced conventional methods in identifying and characterizing bacterial pathogens. We characterized 23 nonserotypeable *Shigella* that biochemically resembled *Shigella* spp. using whole genome sequencing.

**Materials & methods:**

Genome sequences were analyzed using online tools based on 16S rRNA, k-mer, *gyr*B sequences and analysis of O-antigen arrangement was done using PATRIC database for species identification. Sequence types, plasmid types, antimicrobial resistance and virulence genes were also investigated.

**Results:**

The SpeciesFinder using 16S rRNA sequences identified only 74% of the isolates, whereas KmerFinder and *gyr*B sequence analysis identified 100% of the isolates to its species level. Antimicrobial resistance, virulence and plasmid incompatibility groups were identified in all the isolates. Sequence types were determined.

**Conclusion:**

This study shows that whole genome sequencing approach for *Shigella* O-antigen analysis has greater discriminative power than other methods using different bioinformatics pipeline for identification of nonserotypeable *Shigella*.

## Lay abstract

Shigellosis is one of the major health concerns in developing countries. *Shigella* spp. that is not serotypeable with available antisera is a hurdle in laboratory diagnosis. Sequencing technology has replaced conventional methods in identifying nonserotypeable *Shigella*. In this study, we characterized 23 nonserotypeable *Shigella* using whole genome sequences. Manual comparison of O-antigen gene arrangement provided definitive results compared with other methods. Development of a database including whole genome sequencing of all known serotypes will help in easy identification of *Shigella* serotypes in the future.

*Shigella* species have been reported to be the major cause of human gastroenteritis in developing countries. This includes *S. dysenteriae, S. flexneri, S. boydii* and *S. sonnei*. The diarrheal disease caused by *Shigella* is highly contagious due to its low infectious dose. Identification of the bacterial species in clinical specimens is crucial for choosing optimal treatment and for infection control measures. Identification of *Shigella* spp. by biochemical characteristics is suboptimal and requires serotyping for complete identification [1]. The variability of O antigen provides the major basis for serotyping of many Gram-negative bacteria, and it is the only antigen used for serotyping of *Shigella* as they lack H and K antigens [2]. Rarely, the genus *Shigella* is difficult to serotype with the available *Shigella* polyvalent and monovalent antisera, which could be due to morphological transition from smooth to rough forms. Rough isolates do not produce O-antigen (which is responsible for serological diversity) due to mutations in one or more of the multiple genes controlling O-antigen synthesis and polymerization [3]. Further, the modification in the *S. flexneri* O-antigen backbone except serotype 6 gives rise to different serotypes due to the addition of glucosyl, O-acetyl or phosphoethanolamine groups to one or more sugars [4].

Several studies have reported that *Shigella* spp. and other closely related species (such as enteroinvasive *E. coli*) share a similar pathogenic mechanism, yet it is essential to discriminate due to their clinical relevance and for outbreak responses. This close relatedness makes biochemical- and serological-based identification difficult. Although various molecular methods like restriction fragment length polymorphism targeting the *rfb* and *fliC* gene, ribotyping, PFGE (pulse field gel electrophoresis), MALDI-TOF MS and PCR-based methods including ERIC (Enterobacterial Repetitive Intergenic Consensus)-PCR have been proposed in the past years, the discrimination between the species is still challenging [5,6].

Recently, a whole-genome sequence (WGS) based analysis using publicly available bioinformatics tool was found to replace traditional methods. WGS analysis shows better discrimination between closely related species and can provide clinically relevant information [7]. The report by Chattway *et al*., showed that the k-mer-based identification approach on whole genome data effectively differentiated *Shigella* from *E. coli* and provided information on phylogenetic relationship [8].

In this study, we studied the whole genome of 23 nontypeable *Shigella* isolates by various *in-silico* methods. Multiple analyses using organism-specific bioinformatics pipeline were performed to resolve the identification difficulty. Additionally, the isolates were investigated for the presence of antimicrobial resistance (AMR) genes, plasmids. Sequence types were also determined.

## Materials & methods

### Bacterial strains

Seven hundred and sixty four *Shigella* isolates were collected during the years 2011–2015 at Christian Medical College, Vellore, India. All the strains were isolated from stool specimens of patients with gastroenteritis. Individual isolates were characterized using standard biochemical tests [9]. Serologic identification was done by slide agglutination test using polyvalent somatic (O) antigen grouping sera, followed by monovalent antisera (Denka, Seiken, Japan) for *Shigella* specific serotype identification. Twenty-three *Shigella* isolates out of 48 *Shigella* spp. that were identified to be nonagglutinable with either poly- or monovalent *Shigella* antisera were randomly selected and included in the study for further characterization.

### Antimicrobial susceptibility testing

All the isolates were tested for antimicrobial susceptibility against the following antibiotics: ampicillin (10 μg), trimethoprim/sulfamethoxazole (1.25/23.75 μg), nalidixic acid (30 μg), norfloxacin (10 μg), cefotaxime (30 μg) and cefixime (5 μg) by disc diffusion method. Results were interpreted using Clinical and Laboratory Standards Institute Guidelines 2015 [10]. Quality control strains used were: *E. coli* ATCC 35218 for ampicillin and *E. coli* ATCC 25922 for rest of the antibiotics.

### Next generation sequencing

The WGS for the study strains were performed using Ion Torrent (PGM, Life Technologies, CA, USA) with 400-bp read chemistry (Life Technologies). Library preparations were performed according to manufacturer's instructions using (Ion Plus Fragment Library Kit; Life Technologies). Genomic library was purified using AMPure beads, and concentrations were determined using the Qubit 3.0 fluorimeter (Invitrogen, Merelbeke, Belgium). Emulsion PCR was performed on pooled libraries (Ion One Touch Hi-Q 400 Template Kit v2 DL Kit; Life Technologies), and template-positive Ion Sphere particles were enriched using Dynabeads Myone streptavidin C1 beads. Finally pooled samples were loaded on an Ion 318 chip for sequencing.

#### Assembly & annotation

The generated whole genome data were assembled *de novo* using AssemblerSPAdes v.5.0.0.0 embedded in Torrent suite server v.5.0.4. The genome sequence was annotated using PATRIC, the bacterial bioinformatics database and analysis resource (www.patricbrc.org), and the NCBI Prokaryotic Genome Automatic Annotation Pipeline (www.ncbi.nlm.nih.gov/genomes/static/Pipeline.html) [11].

#### Downstream genome analysis

The whole genome data were analyzed using open access tools at Center for Genomic Epidemiology (CGE) web-based server. Sequence types for the study isolates were determined using multilocus sequence typing 1.8 tool (MLST 1.8) (https://cge.cbs.dtu.dk//services/MLST/) [12], AMR and virulence genes were identified using ResFinder 2.1 (https://cge.cbs.dtu.dk//services/ResFinder/) [13] and VirulenceFinder 1.5 (https://cge.cbs.dtu.dk//services/VirulenceFinder/) [14] with the 90% threshold for identity and with 60% of minimum length coverage. Presence of plasmids were analyzed using PlasmidFinder 1.3 (https://cge.cbs.dtu.dk//services/PlasmidFinder/) [15] with 95% threshold for identity.

### WGS analysis for identification of nonserotypeable *Shigella*

#### Species identification using 16S rRNA sequence analysis

SpeciesFinder 1.0 predicts species based on their 16S rRNA gene, where the assembled genome sequences will be aligned against the16S rRNA sequences from the database using the default BLAST algorithm (https://cge.cbs.dtu.dk/services/SpeciesFinder/) [16]. The best BLAST hit was chosen based on the query coverage, % identity, number of mismatches and number of gaps in the alignments.

#### k-mer-based species identification

This method finds the unique k-mers in the input sequence and predicts species based on the number of overlapping k-mers, that is, 16-mers, between the query genome and genomes in a reference database. The prediction was made at which it has the highest number of 16-mers in common despite of position. The program ran with the ‘winner takes it all’ scoring method (https://cge.cbs.dtu.dk/services/KmerFinder/) [7].

#### gyrB sequence based species identification

*Shigella* genomes were analyzed for *gyr*B gene sequences to classify closely related species. The query *gyr*B sequences obtained from WGS were manually BLAST matched using NCBI pipeline [17].

#### Manual serotype identification using O-antigen gene cluster

The number of genes in O-antigen clusters varies and strains of different serotypes can show completely different gene sets. The sequential arrangements of O-antigen genes which are usually bordered by *gal*F and *gnd* genes were compared manually with those available as *Shigella* WGS database in the PATRIC database [18].

#### Phylogenetic analysis

Phylogenetic analysis was performed for *gyr*B sequences of the isolates on the Phylogeny.fr platform. Sequences were aligned with MUSCLE (v3.8.31) with the default settings. After alignment, gaps and poorly aligned sequences were removed with Gblocks (v0.91b) using the following parameters: minimum length of a block after gap cleaning: ten, no gap positions were allowed in the final alignment, all segments with contiguous nonconserved positions bigger than eight were rejected and minimum number of sequences for a flank position: 85%.

The phylogenetic tree was reconstructed using the maximum likelihood method implemented in the PhyML program (v3.1/3.0 aLRT). The HKY85 substitution model was selected assuming an estimated proportion of invariant sites (of 0.910) and four γ-distributed rate categories to account for rate heterogeneity across sites. The γ-shape parameter was estimated directly from the data (γ = 95.586). Reliability for internal branch was assessed using the aLRT test (SH-Like). Graphical representation and edition of the phylogenetic tree were performed with TreeDyn (v198.3) [19,20].

## Results

**Figure 1.**
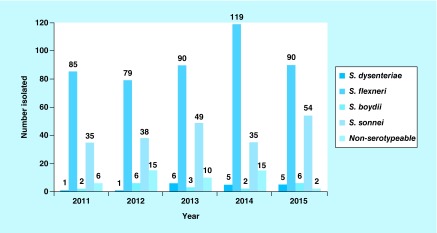
**Serogroup distribution of *Shigella* from feces specimens over 5 years (2011–2015).**

From 2011 to 2015, a total of 716 *Shigella* species with known serotypes were isolated with *S. flexneri* the predominant serogroup followed by *S. sonnei* (Figure 1).

## AMR profile

**Figure 2.**
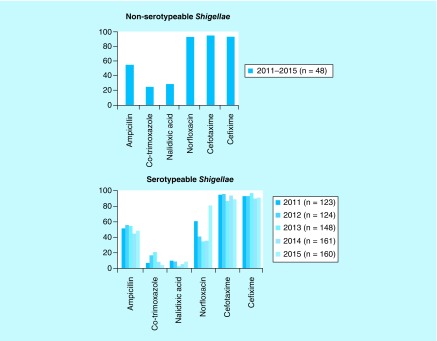
**Antimicrobial susceptibility trend of serotypeable and nonserotypeable *Shigella* spp. over 5 years (2011–2015).**

Among the selected isolates 48% were found to be multidrug resistant. The isolates showed 69% resistance to nalidixic acid followed by trimethoprim/sulfamethoxazole 66%, ampicillin 52%, cefotaxime 17%, norfloxacin 13% and cefixime 4%, respectively. The antimicrobial susceptibility trends of nonserotypeable and typeable *Shigella* spp. over the past 5 years are presented in Figure 2.

## Genome analysis of nonserotypeable *Shigella*

### SpeciesFinder

SpeciesFinder predicts the prokaryotic species based on the 16S rRNA gene similarity with the known reference sequence. This method identified eleven isolates (FC1, FC 2, FC6–FC9, FC14–FC17 and FC20) as *S. boydii*, three isolates (FC10–FC12) as *S. flexneri* but failed to identify the species for six isolates (FC3–FC5, FC13, FC18 and FC19). Further three isolates (FC21–FC23) were identified as *E. coli* (Table 1).

**Table 1.** **Comparative analysis of SpeciesFinder, KmerFinder, *gyr*B sequence and O-antigen gene sequence based identification of nonserotypeable *Shigella*.**

**Isolate ID**	**Manual analysis using PATRIC**	**Center for Genomic Epidemiology**		**BLAST *gyr*B sequence analysis**	**Sequence types**
		**SpeciesFinder**	**KmerFinder**		
FC1	*S. boydii*	*S. boydii*	*S. boydii*	*S. boydii*	145

FC2	*S. boydii*	*S. boydii*	*S. boydii*	*S. boydii*	145

FC3	*S. boydii*	Matching failed	*S. boydii*	*S. boydii*	243

FC4	*S. boydii*	Matching failed	*S. boydii*	*S. boydii*	145

FC5	*S. boydii*	Matching failed	*S. boydii*	*S. boydii*	145

FC6	*S. boydii*	*S. boydii*	*S. boydii*	*S. boydii*	145

FC7	*S. boydii*	*S. boydii*	*S. boydii*	*S. boydii*	145

FC8	*S. boydii*	*S. boydii*	*S. boydii*	*S. boydii*	145

FC9	*S. boydii*	*S. boydii*	*S. boydii*	*S. boydii*	145

FC10	*S. flexneri 2a*	*S. flexneri*	*S. flexneri*	*S. flexneri*	245

FC11	*S. flexneri 2a*	*S. flexneri*	*S. flexneri*	*S. flexneri*	245

FC12	*S. flexneri 2a*	*S. flexneri*	*S. flexneri*	*S. flexneri*	245

FC13	*S. dysenteriae*	Matching failed	*S. boydii*	*S. boydii*^†^	148

FC14	*S. dysenteriae*	*S. boydii*	*S. boydii*	*S. boydii*^†^	148

FC15	*S. dysenteriae*	*S. boydii*	*S. boydii*	*S. boydii*^†^	148

FC16	*S. dysenteriae*	*S. boydii*	*S. boydii*	*S. boydii*^†^	148

FC17	*S. dysenteriae*	*S. boydii*	*S. boydii*	*S. boydii*^†^	148

FC18	–	Matching failed	*S. boydii*	*S. boydii*	243
FC19	–	Matching failed	*S. boydii*	*S. boydii*^†^	148

FC20	–	*S. boydii*	*S. boydii*	*S. boydii*^†^	148

FC21	–	*E. coli*	*E. coli*	*E. coli*	6199

FC22	–	*E. coli*	*E. coli*	*E. coli*	6199

FC23	–	*E. coli*	*E. coli*	*E. coli*	6270

^†^Isolates with only 99% identity.

### KmerFinder

KmerFinder identified the isolates (FC1–FC9, FC13–FC20) as *S. boydii* and isolates FC10, FC11 and FC12 as *S. flexneri*. The three isolates (FC21, 22 and 23) showed similar results as that of SpeciesFinder, by k-mer method and were identified as *E. coli*. (Table 1).

### GyrB sequence based species identification

This method targets *gyrB* gene that encode the subunit B protein of DNA gyrase (topoisomerase type II protein). *gyr*B gene sequences has greater divergence values between the closely related species. BLAST matching the query sequence resulted in identification of the species based on sequence similarity. The results of *gyr*B gene sequence analysis matches with that of KmerFinder as shown in Table 1. But this method identified seven isolates as *S. boydii* only with 99% identity to the reference genome available in the NCBI database.

### Manual serotype identification by O-antigen gene analysis

The genome sequence analysis revealed that the O-antigen structure of the isolates FC1–FC 9 matched with the reference strain *S. boydii* in the database. Isolates FC10–FC12 were found to be *S. flexneri* 2a, and was concordant with all methods. In contrast, five isolates (FC13–FC17) had O-antigen structure similar to *S. dysenteriae* through manual comparison but was identified as *S. boydii* by other three methods. However, the O-antigen gene arrangement of five isolates (FC18–FC22) did not match with any of the reference *Shigella* sequence in the PATRIC database.

### MLST/AMR/virulence identification

MLST reveals the sequence type of the isolates based on allelic profile. The sequence types of the isolates were determined using *E. coli* MLST database [23]. *Shigella boydii* isolates (n = 9) were found to have two sequence types, ST145 and ST243 (Table 1). Five isolates identified as *S. dysenteriae* belong to ST148, and three *S. flexneri* isolates were identified as ST245.

Acquired AMR genes were identified in all except four isolates (FC4, 5, 11 and 23). The virulence genes responsible for *Shigella* pathogenesis are located in the chromosome and in the invasion (inv) plasmid. Multiple virulence factors were identified in 22 isolates using VirulenceFinder tool and are given in Table 2. Plasmid analysis revealed the presence of various incompatibility groups among the isolates, of which IncFII was the most common type (n = 19). Five isolates (FC4, FC5, FC10, FC12 and FC23) were found to have either AMR genes or plasmids (Table 2) and one isolate (FC11) neither had any AMR genes nor plasmids.

**Table 2.** **Genomic characteristics of nonserotypeable *Shigella* (n = 23).**

**Isolate ID**	**Total size (bp)**	**Antimicrobial resistance profile**	**ResFinder**	**VirulenceFinder**	**PlasmidFinder**	**Accession**
FC1	4511440	SXT-NAL	*str*A, *str*B, *aadA1, sul2, dfrA1*	*iha, sen*B, *ipa*D, *vir*F	IncFII	MDDI00000000

FC2	4669256	AMP-SXT	*str*A, *str*B, *bla*TEM1B, *qnr*S1, *sul2, tet*A, *dfrA14*	*vir*F, *ipa*D, *sen*B, *ipa*H, *iha*	IncFII, IncFIB	MDDH00000000

FC3	4553246	SXT-NAL-FIX	*aadA1, sul1, tet*A, *dfrA1, dfrA4*	*vir*F, *cap*U, *ipa*D, *ipf*A, *sen*B, *iha*	IncA/C2, IncFII	MDGW00000000

FC4	4561671	–	–	*iha, cap*U, *ipa*D, *sig*A,*sen*B, virF	IncFII	MDJL00000000

FC5	4579536	AMP-SXT-NAL	–	*iha, cap*U, *sig*A, *ipa*D, *sen*B, *vir*F	IncFII	MIIV00000000

FC6	4556438	AMP-SXT	*str*A, *str*B, *bla*TEM-1B, *qnr*S1, *sul2, tet*A, *dfrA14*	*iha, cap*U, *sig*A, *ipa*D, *sen*B, *vir*F	IncFII, IncFIB	MINP00000000

FC7	4514218	SXT-NAL-NOR	*aadA1, dfrA1*	*sen*B, *ipa*D, *iha, vir*F, *cap*U, *sig*A	IncFII	MINQ00000000

FC8	4512137	SXT	*aadA1, dfrA1*	*sen*B, *ipa*D, *vir*F, *iha, cap*U, *sig*A	IncFII	MINR00000000

FC9	4622022	AMP-SXT-NAL^†^	*str*A.*str*B, *bla*TEM-1B, *qnr*S1, *sul2, dfrA14*	*iha, cap*U, *sig*A, *ipa*D, *sen*B, *vir*F	IncFII, IncFIB	MINU00000000

FC10	4378269	AMP-SXT-NAL-NOR^†^	*str*A, *str*B, *aadA1, bla*OXA-1, *sul2, tet*B, *dfrA1*	*ipf*A, *pic, sig*A	–	MDJJ00000000

FC11	4309816	AMP-SXT	–	*ipf*A	–	MECX00000000

FC12	4411934	AMP-SXT-NAL-NOR^†^	*str*A, *str*B, *bla*OXA1, *sul2, tet*B, *dfrA1*	*ipa*H, *ipf*A, *pic, sig*A	–	MDJI00000000

FC13	4558192	NAL-TAX	*str*A, *str*B, *aadA1, sul2, tet*B, *dfrA1*	*cap*U, *vir*F, *ipa*D, *sig*A, *sen*B, *ipf*A, *iha*	IncFII	MECW00000000

FC14	4561234	SXT	*aadA1, bla*OXA-1, *tet*B, *dfrA1*	*cap*U, *ipa*D, *sig*A, *sen*B, *iha, ipf*A, *vir*F	IncFII	MIIX00000000

FC15	4655758	NAL	*tet*B, *dfrA1*	*cap*U, *ipa*D, *sen*B, *ipf*A, *sig*A, *iha, vir*F	IncFII	MIIY00000000

FC16	4575582	AMP-NAL-TAX	*aadA1, bla*OXA-1, *tet*B, *dfrA1*	*cap*U, *vir*F, *ipa*D, *sig*A, *sen*B, *ipf*A, *iha*	IncFII	MINS00000000

FC17	4561447	AMP-NAL-TAX	*aadA1, bla*OXA-1, *tet*B, *dfrA1*	*cap*U, *ipa*D, *iha, sig*A,*sen*B, *ipf*A, *vir*F	IncFII	MINT00000000

FC18	4502544	AMP-SXT-NAL	*str*A, *str*B, *aadA1, bla*TEM-1B, *qnr*S1, *sul2, dfrA1*	*ipf*A, *cap*U, *ipa*D, *vir*F, *sen*B, *iha, sig*A	IncN, IncFII	MDJK00000000

FC19	4458457	AMP-SXT-NAL	*str*A, *str*B, *bla*TEM-1B, *qnr*S1, *sul2, dfrA14*	*cap*U, *ipa*D, *sig*A,*sen*B, *iha, ipf*A, *vir*F	IncFII, IncFIB	MECT00000000

FC20	4651491	AMP-SXT-NAL	*str*A, *str*B, *aadA1, bla*OXA-1, *sul2, tet*B, *dfrA1*	*cap*U, *ipa*D, *iha, sig*A, *sen*B, *ipf*A, *vir*F	IncFII	MINV00000000

FC21	5432092	–	*dfrA5, sul1*	*sen*B, *sat, cap*U, *iha, ipf*A	IncFII, Col(BS512), ColpVC, IncFIB, IncB/O/K/Z	MDES00000000

FC22	5416583	NAL-TAX	*dfrA5, sul1*	*sen*B, *iha, sat, cap*U, *ipf*A	IncFII, IncFIB, Col(BS512), IncB/O/K/Z. Col156	MDDJ00000000

FC23	4664034	NAL	–	–	IncX1	MECV00000000

^†^Intermediate susceptible.

### Phylogenetic analysis of *gyr*B gene

**Figure 3.**
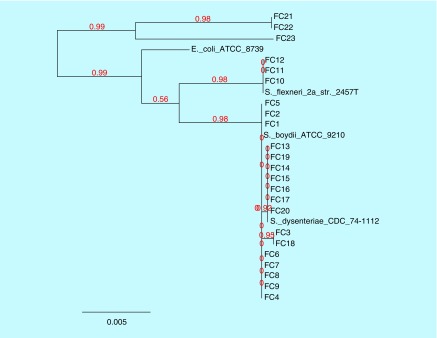
**Phylogenetic tree was constructed using maximum likelihood method of nucleotide sequences of *gyr*B genes for the study isolates.**

Along with the study isolates, known *gyr*B sequence data of *S. dysenteriae, S. boydii, S. flexneri* and *E. coli* were included to deduce the relationship between isolates. Within these three strains, FC21, 22 and 23, were *E. coli*, while other strains were *Shigella* spp. which is related to their common ancestor *E. coli*. FC10, 11 and 12 form a branch and matches with *S. flexneri*, while others are related to *S. boydii* and formed a separate clade. In this clade, 8 strains (FC1, 2, 4, 5, 6, 7, 8 and 9) were found to be *S. boydii*, from which two branches were evolved. One branch had seven strains (FC13, 14, 15, 16, 17, 19 and 20) with 92% similarity with *S. boydii* and the other had two strains (FC3 and 18) with 95% similarity with *S. dysenteriae*. The phylogenetic tree for the isolates based on their *gyr*B gene sequences is shown in the Figure 3.

## Discussion

Although *Shigella* and *E. coli* share common characteristics there is often a clinical or public health need to differentiate these pathogens as they have different entities in epidemiology and clinical medicine. The incidence of new variants of *Shigella* serotype which do not agglutinate with commercially available antisera is increasing [21]. Studies on molecular characterization of such isolates are very rare especially in developing countries with a lower resource setting. In one such study, research from Kolkata, India, characterized 13 provisional serovars of *Shigella* with respect to their AMR, plasmids, virulence genes and PFGE profiles [21].

Sequence types identified in the current study are reported earlier. Previous studies have shown that the sequence type ST245 was found in various *S. flexneri* serotypes (1a, 1b, 2a, 2b, 3a, 3b, 3c, 4a, 4b, 4c and X) [22]. ST243 was recognized in *S. flexneri* serotypes 6 and 6a, *S. boydii* 1, 3, 6, 8, 10 and18 and *S. dysenteriae* 5 and 7. Several STs may be present within a serotype, therefore categorizing the serotypes based on the ST's is not possible [22].

Previous studies from Iran and other countries observed that the tetracycline resistance was seen in the majority of the clinical *Shigella* strains due to the development of intrinsic resistance to this antibiotic. Shahsavan *et al*., reported the MLST of multidrug resistant *Shigella* spp. and found that ampicillin resistance was frequently observed in ST245 and tetracycline susceptibility in *S. flexneri* ST145 strain. This suggests that isolates with different sequence types correspond to specific AMR patterns. Regular evaluation of ST and AMR data will help to treat shigellosis caused by resistant strains [23]. In this study, eight isolates identified as *S. boydii* belong to ST145. Novel sequence types were obtained for *E. coli* (ST6199 and ST6270) in this study and have not been reported earlier.

Genome annotation results showed more than 50-times coverage for all the isolates. The genome sequences were analyzed *in-silico* by different methods using various web-based tools for species identification. SpeciesFinder, which is based on the 16S rRNA gene, had shown lesser resolution than the other methods discussed in this study, which identified only 17 isolates to species level. This could be due to substantial intergene variation and the fact that 16S rRNA gene corresponds to 0.1% of the coding part of a microbial genome (Table 3). Bacterial identification using 16S rRNA sequencing was reported to have limitations such as its inability to distinguish atypical *E. coli* from *Shigella* spp. The sequence similarities of *S. flexneri, S. sonnei* and *S. boydii* with *E. coli* were reported to be 99.8, 99.9 and 99.7%, respectively [24]. This shows that the species identification by 16S rRNA sequences is not reliable and accurate.

**Table 3.** **16S rRNA sequence similarity within *Shigella* species.**

**Organism**	***S. dysenteriae***	***S. boydii***	***S. sonnei***	***S. flexneri***	***S. flexneri 5a***
*S. dysenteriae*	100				

*S. boydii*	98.72	100			

*S. sonnei*	98.79	99.4	100		

*S. flexneri*	99.06	99.66	99.73	100	

*S. flexneri 5a*	98.86	99.47	99.68	99.8	100

KmerFinder identified all the isolates to species level. The method predicts the species based on the closest match to the isolate in the k-mer database. Among the tested isolates, FC17 and 12 isolates results were in concordance with the SpeciesFinder and PATRIC respectively.

The *gyr*B gene sequence analysis presented better results when compared with 16S rRNA analysis; this is in accordance with the previous study, where *gyr*B gene analysis was found to be effective in classifying closely related species [17]. The results obtained by this method matched with KmerFinder results. This can be explained as the rate of genetic divergence of *gyr*B sequences differed greatly from 16S rRNA sequence and have four- to tenfold increase in the length of branches between closely related species of *Shigella* and *E. coli*. The percent divergences of *E. coli* from *S. sonnei, S. flexneri* and *S. boydii* were 1.9, 2.3 and 2.0%, respectively, indicating the reliability of *gyr*B gene sequence analysis method than 16S rRNA analysis [24]. However, this method identified few isolates with only 99% identity and does not provide accurate (100%) identification results.

Analysis of O-antigen gene cluster arrangement using PATRIC revealed the isolates FC18–FC23 did not match with any known *Shigella* sequences in the PATRIC database, which could be due to limitations inherent to the database. However, the rest of the isolates showed similar results to those of SpeciesFinder, KmerFinder and *gyr*B sequence analysis methods.

Five isolates (FC13–FC17) was identified as *S. dysenteriae* through manual comparison of O-antigen using PATRIC but were misidentified as *S. boydii* by the other three methods. Traditionally, *S. boydii* and *S. dysenteriae* are physiologically similar but were differentiated biochemically by the mannitol test and these two species can be found within the same STs, which shows their close phylogenetic relationship.

Overall, SpeciesFinder, based on the 16S rRNA gene analysis, showed poor performance, which can identify only 74% of the isolates up to species level. KmerFinder and *gyr*B sequence analysis both identified 100% of the isolates to the species level despite its accuracy.

Occasionally, O-antigen modification within the species affects the serotyping assay and makes the strain untypeable. The modification is widely seen in *S. flexneri* serotypes (1a, 1b, 2a, 5a and Y) as these have bacteriophage-mediated serotype conversion [4]. Consequently the laboratory identification of *Shigella* by agglutination method becomes difficult with commercially available polyvalent-antisera and may not cover all possible epitopes of the *Shigella* O-antigen. Another problem is due to high genetic similarity between *Shigella* and *E. coli* and in fact that majority of *Shigella* O antigens cross-react serologically with some strains of *E. coli* O antigen [2]. It was found that cross-reacting strains of *Shigella* spp. and *E. coli* may express shared lipopolysaccharide epitopes yet their lipopolysaccharide structures are not identical [25]. Therefore, differentiation between *Shigella* and cross-reacting enteroinvasive *E. coli* strains are often difficult as both show similar biochemical traits and can cause dysentery using the same mode of invasion [26].

In contrast to the earlier study by Dutta *et al*. [21] who demonstrated that multidrug resistance was reported rarely in the provisional serovars of *Shigella* isolates, most of our study isolates were found resistant to first-line antibiotics like ampicillin, trimethoprim/sulfamethoxazole and nalidixic acid but were highly susceptible to second-generation quinolone (norfloxacin) and third-generation cephalosporin (cefotaxime and cefixime). The nontypeable *Shigella* isolates from our setting over the last 5 years showed susceptibility of around 90% to norfloxacin, cefotaxime and cefixime in contrast to typeable *Shigella* isolates but had similar resistance profile for ampicillin. Serotypeable isolates were highly resistant to co-trimoxazole and nalidixic acid compared with nontypeable isolates.

Further, the study isolates were found to harbor AMR genes through sequence analysis and the results correlate with the phenotypic resistance profile except for three isolates (FC5, FC11 and FC23). One isolate (FC21) does not show any resistance phenotypically but had AMR genes.

Molecular typing of nonserotypeable *Shigella* is important as this species represents the major causes of bacterial diarrhea in developing countries. This study shows that some limitations such as inability to distinguish closely related species were observed with the 16S rRNA sequence method, but this can be overcome by the k-mer approach and *gyr*B gene analysis. However, the manual comparison of O-antigen gene arrangement provided considerable and more definitive results than the k-mer and *gyr*B method in identifying the species although it could not identify a few isolates due to the lack of genome sequences of all *Shigella* serotypes in the database. This study underlines the need for genome sequences of all known *Shigella* serotypes reported till date, for detailed understanding of the species with enhanced resolution.

## Limitations

A reference database including O-antigen sequences of all known serotypes of *Shigella* spp. was not available. Manual comparison of O-antigen arrangements is error prone; however, utmost care was taken to avoid such errors.

## Conclusion & future perspective

The O-antigen determination of clinical *Shigella* isolates is essential for diagnostic and epidemiologic purpose. Knowledge on the distribution of different *Shigella* serotypes remains important as humans demonstrate only serotype-specific immunity. Therefore, the characterization of isolates using WGS could provide better results and have greater discriminative power compared with other commonly used methods. Development of a database including WGS of all known *Shigella* serotypes is required for future identification and comparison of nontypeable *Shigella*. Also, an SNP-based phylogenetic analysis will be a supporting evidence for *Shigella* serotype diversification.

**Summary points**

**Background**

- Identification of the bacterial species in clinical specimens is crucial for choosing optimal treatment and for infection control measures.
- The discrimination between closely related species is still challenging. This close relatedness makes the biochemical and serological based identification difficult.
- Recently, whole genome sequencing technology has replaced the conventional methods in identifying and characterizing the bacterial pathogens.
- Here, we studied the whole genome of 23 nonserotypeable *Shigella* isolates to resolve the identification difficulty.
- Overall, SpeciesFinder, based on the 16S rRNA gene, had the poorest performance, which identifies only 74% of the isolates up to its species level.
- Whereas KmerFinder and *gyr*B sequence analysis, both had the highest performance and identify 100% of the isolates to the species level.
- The manual comparison of O-antigen gene arrangement was considered to be the reliable method for identifying nonserotypeable *Shigella*.

**Conclusion**

- Whole genome sequencing was found to have greater discriminative power compared with other commonly used methods.
- Also knowledge on the distribution of different *Shigella* serotypes remains important as humans demonstrate serotype specific immunity.

## References

[1] Grimont F, Lejay-Collin M, Talukder KA (2007). Identification of a group of *Shigella*-like isolates as *Shigella boydii* 20. *J. Med. Microbiol.*.

[2] Liu B, Knirel YA, Feng L (2008). Structure and genetics of *Shigella* O antigens. *FEMS Microbiol. Rev.*.

[3] Coimbra RS, Grimont F, Grimont PAD (1999). Identification of *Shigella* serotypes by restriction of amplified O-antigen gene cluster. *Res. Microbiol.*.

[4] Jakhetia R, Marri A, Stahle J, Widmalm G, Verma NK (2014). Serotype-conversion in *Shigella flexneri*: identification of a novel bacteriophage, Sf101, from a serotype 7. *BMC Genomics*.

[5] Muthuirulandi Sethuvel DP, Devanga Ragupathi NK, Anandan S, Walia K, Veeraraghavan B (2017). Molecular diagnosis of non-serotypeable *Shigella* spp.: problems and prospects. *J. Med. Microbiol.*.

[6] Pettengill EA, Pettengill JB, Binet R (2016). Phylogenetic analyses of *Shigella* and enteroinvasive *Escherichia coli* for the identification of molecular epidemiological markers: whole-genome comparative analysis does not support distinct genera designation. *Front. Microbiol.*.

[7] Hasman H, Saputra D, Sicheritz-Ponten T (2014). Rapid whole-genome sequencing for detection and characterization of microorganisms directly from clinical samples. *J. Clin. Microbiol.*.

[8] Chattaway MA, Schaefer U, Tewolde R, Dallman TJ, Jenkins C (2016). Identification of *Escherichia coli* and Shigella species from whole genome sequences. *J. Clin. Microbiol.*.

[9] Koneman EW, Allen SD, Janda WM, Schreckenberger PC, Winn WC (1997). *Colour Atlas and Textbook of Diagnostic Microbiology (5th Edition)*.

[10] CLSI, Clinical and Laboratory Standards Institute (2015). *Performance Standards for Antimicrobial Susceptibility Testing; Twenty-fourth Informational Supplement M100-S25*.

[11] Tatusova T, DiCuccio M, Badretdin A (2016). NCBI prokaryotic genome annotation pipeline. *Nucleic Acids Res.*.

[12] Larsen MV, Cosentino S, Rasmussen S (2012). Multilocus sequence typing of total genome sequenced bacteria. *J. Clin. Micobiol.*.

[13] Zankari E, Hasman H, Cosentino S (2012). Identification of acquired antimicrobial resistance genes. *J. Antimicrob. Chemother.*.

[14] Joensen KG, Scheutz F, Lund O (2014). Real-time whole-genome sequencing for routine typing, surveillance, and outbreak detection of verotoxigenic *Escherichia coli*. *J. Clin. Micobiol.*.

[15] Carattoli A, Zankari E, Garcia-Fernandez A (2014). Plasmid Finder and pMLST: *in silico* detection and typing of plasmids. *Antimicrob. Agents Chemother.*.

[16] Larsen MV, Cosentino S, Lukjancenko O (2014). Benchmarking of methods for genomic taxonomy. *J. Clin. Microbiol.*.

[17] Nochi Z, Sahebekhtiari N, Kharaziha P (2009). Comparison of 16S rRNA, 23S rRNA and *gyrB* genes sequences in phylogenetic relationships of Shigella isolates from Iran. *Ann. Microbiol.*.

[18] Wattam AR, Abraham D, Dalay O (2014). PATRIC, the bacterial bioinformatics database and analysis resource. *Nucl. Acids Res.*.

[19] Dereeper A, Guignon V, Blanc G (2008). Phylogeny.fr: robust phylogenetic analysis for the non-specialist. *Nucleic Acids Res.*.

[20] Dereeper A, Audic S, Claverie JM, Blanc G (2010). Blast-explorer helps you building datasets for phylogenetic analysis. *BMC Evol. Biol.*.

[21] Dutta S, Jain P, Nandy S, Matsushita S, Yoshida S (2014). Molecular characterization of serologically atypical provisional serovars of *Shigella* isolates from Kolkata, India. *J. Med. Microbiol.*.

[22] Choi SY, Jeon YS, Lee JH (2007). Multilocus sequence typing analysis of *Shigella flexneri* isolates collected in Asian countries. *J. Med. Microbiol.*.

[23] Shahsavan S, Nobakht M, Rastegar-Lari A, Owlia P, Bakhshi B (2016). Multi-locus sequence type analysis of *Shigella* spp. isolates from Tehran, Iran. *Iran J. Microbiol.*.

[24] Fukushima M, Kakinuma K, Kawaguchi R (2002). Phylogenetic analysis of *Salmonella, Shigella*, and *Escherichia coli* strains on the basis of the gyrB gene sequence. *J. Clin. Microbiol.*.

[25] Chart H, Daniel RMA, Cheasty T (2009). The expression of lipopolysaccharide by strains of *Shigella dysenteriae, Shigella flexneri* and *Shigella boydii* and their cross-reacting strains of *Escherichia coli*. *FEMS Microbiol. Lett.*.

[26] Ud-Din A, Wahid S (2015). Relationship among *Shigella* spp. and enteroinvasive *Escherichia coli* (EIEC) and their differentiation. *Braz. J. Microbiol.*.

